# Development of a menopause-specific questionnaire: Content and face validation using a modified Delphi technique

**DOI:** 10.1177/17455057261476566

**Published:** 2026-08-04

**Authors:** Amanda Calvin, Camilla Udo, Kerstin Erlandsson

**Affiliations:** 1 School of Health and Welfare, 101092 Dalarna University, Falun, Sweden

**Keywords:** menopause, Delphi method, questionnaire development, informed choice, health literacy, menopausal hormone therapy, women’s health

## Abstract

**Background:**

Menopause is a universal life stage, yet many women face persistent knowledge gaps, misconceptions, and unequal access to care. Existing instruments assess isolated aspects of menopause but do not capture women’s access to the conditions necessary for making informed choices about their health.

**Objective:**

The aim of this study was to develop and establish content and face validity of a menopause-specific questionnaire designed to assess women’s access to the preconditions for making informed choices about their health during the menopausal transition and beyond, using a modified Delphi technique.

**Design:**

A modified Delphi study.

**Methods:**

Five Delphi rounds were conducted in 2025 involving an expert group (n = 9), a Public and Patient Involvement (PPI) group (n = 7), and a pilot test group (n = 48). Experts reviewed and refined an initial 39-item draft, the PPI group assessed face validity and readability, and experts conducted item prioritization using predefined consensus thresholds. Pilot testing evaluated usability and respondent burden. Final consensus required ≥80% expert agreement. The study followed CREDES guidelines.

**Results:**

Following content and face validation, the final instrument comprised 29 items. Expert review informed revisions to terminology, neutrality of menopausal hormone therapy items, and language accessibility. PPI contributors highlighted readability challenges and supported clearer wording. Nine items were removed, five merged into two, and one item added. Pilot testing confirmed acceptable usability and respondent burden. Final expert consensus reached 87.5%.

**Conclusion:**

A consensus-based, user-informed questionnaire was developed, following content and face validation, to assess women’s access to conditions for informed choice during menopause. The instrument may support the identification of barriers and facilitators to informed decision-making in menopause-related care and research.

## Plain language summary

Menopause is a major health transition, yet many women navigate it without clear, balanced information about their symptoms and the options available to them. This can make informed choices about their health difficult. While several questionnaires assess general health knowledge or decision-making, none has been developed specifically to capture whether women have the information and support they need to make informed choices in relation to menopause. Working alongside health professionals — doctors, midwives, and a women’s health advocate — and women with personal experience of menopause, we developed a questionnaire. Women and professionals reviewed each question for clarity and relevance, and a further group tested the final version. The result is a 29-item questionnaire, developed through a structured, consensus-based process that reflects both clinical expertise and women’s lived experience. It will be used in a survey across Sweden in 2026. The questionnaire aims to identify where women lack adequate information and support, so that clinical care, health education, and policy can better meet their needs.

## Introduction

Menopause is a natural and inevitable part of life for individuals born with ovaries. Despite its universality, menopause remains a life phase often surrounded by ignorance, misconceptions, and a lack of adequate support from healthcare systems.^1^ For many, it involves a complex interplay of physical, emotional, and social changes that may significantly impact women’s overall quality of life, social relationships and working life.^2–4^ This study uses the word menopause broadly, encompassing the stages of perimenopause, menopause, and postmenopause, unless otherwise specified. Perimenopause, with its hormonal fluctuations leading up to menopause, typically lasts 4–7 years, though hormonal changes associated with reproductive aging may begin up to a decade before the final menstrual period.^5,6^ The median age at menopause in high-income countries is 52 years, with a normal range of 45–55 years.^5^ Given this variability, women at the lower end of the normal range may begin experiencing hormonal changes as early as their mid-thirties. While acknowledging that not all individuals experiencing menopause identify as women, the term ‘women’ is used throughout this text since many going through menopause identify as such.

In 2021, the Swedish National Board of Health and Welfare^7^ identified knowledge gaps among women as well as among healthcare professionals. They also identified geographical and educational differences in access to care. The consequences of these gaps include delayed care-seeking and underutilization of effective therapies. In response to these gaps, a Swedish research team has initiated a doctoral project that seeks to map women’s knowledge, attitudes, and access to care during the menopausal transition entitled *“Bridging the Menopause Gap: Exploring Experiences, Knowledge, Perceptions, and Healthcare Support”.* The overarching goal of the doctoral project is to improve conditions for individualized and equitable menopausal care, using the concept of informed choice as a central analytical framework guiding the project’s design and research questions.

Healthcare in Sweden is obliged to provide person-centered care^8–11^ and inform the patient about the best available care options to enable for the patient to make informed choices. The concept of informed choice originated in midwifery feminist research; midwives and their clients took the view that when women understand their own bodies and their clinical options, they could make informed choices about their care regarding pregnancy and birth.^12^ The concept consists of three equally important dimensions: access to knowledge, easy access to healthcare and treatment, and freedom from myths, stigmas, and misinformation.^13–15^ These dimensions are highly relevant in the context of menopause and menopausal hormone therapy (MHT), where persistent concerns, particularly the fear of breast cancer following the WHI study,^16^ continue to shape public and professional attitudes.^17^ Health literacy is a central precondition for informed choice. Sorensen et al.^18^ describe health literacy as the knowledge, motivation, and competencies needed to access, understand, appraise, and apply health information. This framework aligns closely with the dimensions of informed choice, which require individuals to be able to locate information, understand it, and evaluate their options. Although “menopausal health literacy” is not yet a broadly established concept, work on menstrual health literacy may illustrate relevant challenges. Critchley et al.^19^ identify structural issues such as stigma, normalization of pain, and inconsistent terminology, all of which hindered menstrual health literacy, comprehension, communication and positive healthcare encounters.

Surveys are a widely used and accessible method for collecting quantitative data from large populations, particularly when the aim is to assess knowledge, attitudes, and decision-making processes without imposing excessive respondent burden^20,21^ Several validated instruments address components relevant to informed choice in healthcare. For example, the Multi-Dimensional Measure of Informed Choice (MMIC) was developed in the context of prenatal screening for Down syndrome and focuses on knowledge, values, and decision implementation.^22^ The Health Literacy Questionnaire (HLQ) provides a broad assessment of health literacy across multiple domains,^23^ while the Decisional Conflict Scale,^24^ including the SURE test,^25^ measures individuals’ confidence and uncertainty when making complex health decisions. In the field of menopause, symptom-focused instruments such as the Menopausal Rating Scale (MRS) are available and commonly used to assess symptom burden.^26^ These instruments address isolated dimensions rather than the full set of preconditions required for informed choice during the menopausal transition. None are designed to capture women’s combined access to relevant knowledge, their ability to appraise information, and their access to supportive healthcare in a menopause-specific context. Despite broad consensus regarding the impact of menopause on women’s quality of life and long-term health, and despite growing public and media attention to menopause,^27^ there remains a lack of structured, validated, and user-informed instruments. Therefore, the aim of this study was to develop and establish content and face validity of a menopause-specific questionnaire designed to assess women’s access to the preconditions for making informed choices about their health during the menopausal transition and beyond, using a modified Delphi technique. These preconditions encompass: (1) access to evidence-based knowledge and information about menopause, symptoms, and treatment options including MHT; (2) easy access to healthcare services, treatment, and follow-up; and (3) freedom from stigma and misinformation.^13,15,28^

## Methods

### Design

A modified Delphi study was conducted in Sweden in 2025. The Delphi method enables structured consensus-building in areas lacking established evidence and is well suited for integrating perspectives from academia, clinical practice, and individuals with lived experience.^29^ To ensure the questionnaire was both scientifically rigorous and grounded in real experience, the process incorporated input from academic and clinical experts, as well as from participants with lived menopausal experience. This participatory approach aligns with contemporary recommendations for person-centered instrument development and enhances the contextual relevance and acceptability of the final tool.^30^

The research team consisted of an interdisciplinary group with expertise in nursing science, midwifery, social science, and women’s health. The study followed CREDES (Conducting and REporting of DElphi Studies) reporting guidelines to ensure methodological rigor and transparency.^31^ To align the instrument development with the study aim, the Delphi design was deliberately modified to incorporate lived experience perspectives and an external pilot test. These modifications strengthened clarity, usability, and acceptability while maintaining clinical accuracy and methodological rigor.

### Participants

The overall Delphi panel consisted of three groups: an expert group (n=9), a Public and Patient Involvement (PPI) group (n=7), and a pilot test group (n=48). Combined demographic information for all participant groups is presented in Table 1.

**Table 1.** Combined demographic information for the Delphi panel.

Variable	Expert group (n = 9)	PPI group (n = 7)	Pilot test group (n = 48)
Geography	Not applicable	Urban (3), Suburban (3), Rural (1)	Urban 65%, Suburban 21%, Rural 10%, non-respondent 4%
Profession/Role	Gynecologists (3), midwives (4), sociologist (1), women’s health advocate (1)	Lived menopause experience	Lived menopause experience
Education level	RNRM (2); RNRM, MSc in sexology (1); RNRM, PhD in Medicine (1); PhD in Social Science (1); Professor, MD, specialized physician with senior academic rank (1); MD, PhD students in gynecology (2); BSc in sports science (1)	MSc (1); BSc (3); vocational healthcare (1); upper secondary school (2)	Upper secondary school 23%; post-secondary 19%; BSc 39%; MSc+ 19%
Country of birth	Not applicable	Sweden (6); outside Sweden (1)	92% Sweden; 8% outside Sweden
Menopause status	Not applicable	Perimenopause (2), Postmenopause (3), Surgical menopause (2)	Premenopause 21% (incl. regular & contraceptive-related unclear), Perimenopause 17%, Postmenopause 52%, Surgical/no uterus 4%, Unclear due to HRT 6%
Clinical experience with menopause	1 year (1); 5 years (1); 10 years (2); >10 years (3) No clinical work (2)	Not applicable	Not applicable
MHT use	Not applicable	Use/used MHT (3), Not used MHT (4)	59% have used or currently use MHT

Abbreviations: BSc, Bachelor of Science; MD, medical doctor; MHT, menopausal hormone therapy; MSc, Master of Science; PhD, Doctor of Philosophy; RNRM, registered nurse & registered midwife.

### Expert group

A total of 17 professional experts were invited to participate in the Expert group. Potential experts were identified through the research team’s network based on their scientific publications on menopause or their established roles in women’s health. A purposive sampling strategy ensured diversity across academic, clinical, and advocacy perspectives. Experts were contacted individually by email with study information and an invitation to participate. Experts were eligible if they had recognized competence in women’s health, menopause research, or clinical management, either through academic qualifications or professional experience.^32^ Twelve experts were initially contacted; six agreed to participate. An additional five invitations resulted in three further participants. Experts were excluded if they lacked documented competence in women’s health, menopause research, or clinical management. To maintain confidentiality, survey responses were collected without requesting identifying information, meaning that individual responses could not be directly traced back to specific participants.

### Public and Patient Involvement (PPI) group

PPI groups are essential for incorporating the lived experiences of the target population and for ensuring that research outcomes are relevant and meaningful.^33^ The PPI group members were recruited through a social media invitation distributed within the larger research project. PPI group members represented varied sociodemographic backgrounds and included women at different stages of menopause. Participants were excluded if they were outside the age range of 35–65, born without ovaries, or did not have sufficient Swedish proficiency.

### Pilot test group

The pilot test group members were external persons, recruited through the PPI group and the expert group. Inclusion criteria were age 35–65, being born with ovaries, and sufficient Swedish proficiency to complete the questionnaire. The rationale for the wide age range, spanning from late premenopause to postmenopause, reflects that hormonal changes associated with reproductive aging may begin up to a decade before the final menstrual period.^6^ Participants were excluded if they were outside the age range of 35–65, born without ovaries, or did not have sufficient Swedish proficiency.

### The Delphi procedures

The first author compiled the initial item pool, invited the Delphi panel members in the three groups and managed the Delphi procedures in all groups, as well as editing throughout the process until the final questionnaire was agreed upon, with support from the entire research team. The Delphi process consisted of five rounds: (1) expert relevance review, (2) PPI relevance and readability review, (3) the expert group conducted item prioritization and ranking using relevance scoring with predefined consensus thresholds, (4) cognitive/usability pilot test, and (5) final consensus which required ≥80% agreement in the expert group.

**Figure 1.**
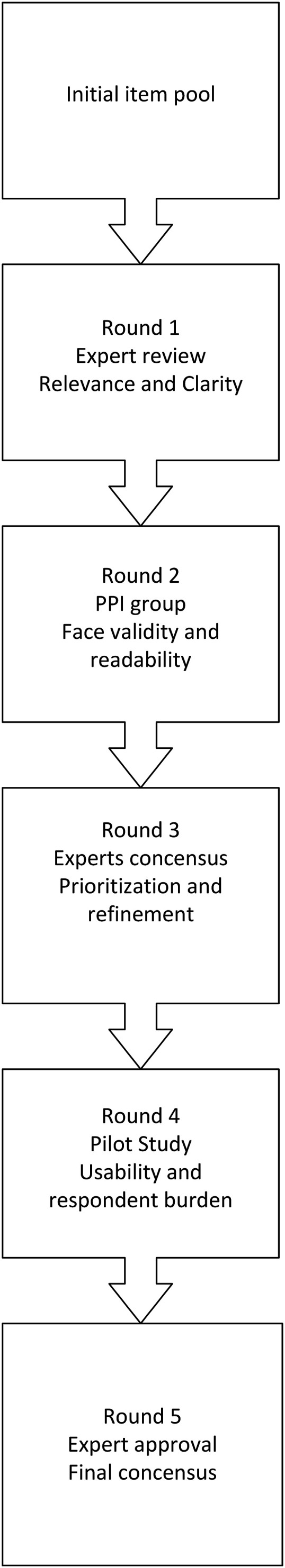
Visualization of the modified Delphi process.

Two planned deviations from a classical Delphi design were incorporated. First, a dedicated PPI round was added to ensure that lived experience perspectives informed item development early in the process. Second, a cognitive and usability pilot test was integrated as Round 4. At the start of rounds 3 and 5, the expert group was provided with anonymized feedback summaries, including thematic summaries of qualitative comments. Feedback was presented in aggregated form only; no individual responses were visible to any participant. These summaries allowed the expert group to revise their views based on group-level feedback while maintaining full anonymity. Quantitative and qualitative findings were integrated in the questionnaire after each round; quantitative ratings guided consensus decisions, while qualitative comments informed item revisions and the wording adjustments carried forward to subsequent rounds. The Delphi process was concluded when predefined consensus thresholds were met and no further substantive revisions were proposed by the expert group. All data collection across Delphi rounds was conducted using Sunet Survey, a secure, GDPR-compliant online platform. The overall structure and sequencing of the modified Delphi process are illustrated in Figure 1. Table 2 summarizes the purpose, participants, key inputs, and outcomes of each Delphi round.

**Table 2.** Overview of the modified Delphi process, rounds, and outcomes.

Delphi round	Participants	Purpose	Key input	Outcome
Round 1	Experts (n=9)	Assess relevance and clarity	Review of 39 items derived from MMIC, HLQ, DCS/SURE	Rewording of 27 items; 2 new items suggested
Round 2	PPI group (n=7)	Face validity & readability	Workshop + online review	Language simplification; terminology support
Round 3	Experts (n=8)	Prioritization & consensus	4-point relevance rating	9 items removed; 5 merged into 2; 1 added
Round 4	Pilot (n=48)	Usability & burden	Completion time + qualitative feedback	1 item removed; MHT section made optional
Round 5	Experts (n=8)	Final approval	Full instrument review	Final 29-item questionnaire approved (87.5%)

Abbreviations: MMIC, Multi-dimensional Measure of Informed Choice; HLQ, Health Literacy Questionnaire; DCS/SURE, Decisional Conflict Scale/Sure of myself, Understanding information, Risk-benefit ratio, Encouragement; PPI, Public and Patient Involvement; MHT, Menopausal Hormone Therapy.

### The initial tool

The initial questionnaire items were developed using items adapted from three validated instruments relevant to informed choice and decision-making: the Multi-dimensional Measure of Informed Choice (MMIC),^22,28,34^ the Health Literacy Questionnaire (HLQ),^23,35^ and the Decisional Conflict Scale including the SURE test (DCS/SURE).^24,25^ These instruments informed the structure, domains, and formulation of items by illustrating how knowledge, attitudes, and confidence in decision-making can be assessed in a systematic way. Based on this guidance, items were adapted and reformulated to reflect a menopause-specific context. Additional items were created by the research team in line with the aim of the study. The first draft questionnaire consisted of 39 items, including 26 derived from MMIC, 8 from HLQ, 1 from DCS/SURE. 4 items were independently developed by the research team, 3 items asked about healthcare preferences and knowledge providers and 1 item asked about overall attitudes towards menopause. Thematically, the Delphi items addressed knowledge (n = 21), attitudes (n = 12), and healthcare access or experiences (n = 6). These items formed the basis for Round 1 of the Delphi process. All items were presented as statements with Likert-scale response options and complemented by free-text fields to allow qualitative feedback. All item-level changes across Delphi rounds are documented in Supplementary Table S1, providing a complete audit trail from the initial 39 items to the final 29-item questionnaire.

#### Round 1: Expert group member’s review of the initial questionnaire draft

The expert group was invited to review the initial 39-item questionnaire. Experts received instructions by e-mails and were given two weeks to complete the assessment online with a provided link. They evaluated each item for clarity and relevance and provided written comments and suggestions for refinement. Experts were also asked to suggest additional items relevant to the domains of informed choice: knowledge, attitudes, and healthcare access in addition to the online survey options.

#### Round 2: Review by the PPI group

The PPI-group was invited to review the questionnaire in a face-to-face workshop. Prior to a workshop session, they received the link to the questionnaire to review independently. During the workshop, the PPI group provided insights on readability, language accessibility, and overall relevance. Additional feedback was delivered through the online survey option. This process supported the evaluation of face validity by ensuring that the questionnaire items were perceived by intended respondents as meaningful, clear, and reflective of their experiences.

#### Round 3: Expert group members prioritization and consensus evaluation

The expert group members received an updated draft of the questionnaire through e-mails alongside a short summary video presenting findings from Rounds 1 and 2. Experts were asked to rate the relevance of each item on a 4 point Likert scale and provide optional written comments. A predefined consensus rule was applied: items receiving low relevance ratings from five or more experts (≥5 of 8) were to be removed.

#### Round 4: Pilot test group

Round 4 involved conducting a pilot test with 48 target users meeting predefined inclusion criteria. The pilot test group completed the questionnaire online and responded to supplementary questions regarding relevance, readability, usability, time and the overall experience of completing the survey. The purpose of this round was to evaluate respondent burden and ensure that the instrument functioned well for target users in practice. Participants also completed demographic questions and the Menopausal Rating Scale (MRS), which will form part of the final instrument in subsequent studies. As this study focused on content and face validation, these data are not reported in the Results section.

#### Round 5: Final expert group approval

In the final Delphi round, the expert group was asked to approve or reject the questionnaire in its entirety. A predefined consensus threshold of ≥80% agreement was applied. Experts who did not approve the questionnaire were required to provide written comments specifying the reasons for non-approval. Two reminder emails were sent out. The Delphi process was concluded once the predefined consensus criterion was met and no further substantive revisions were proposed.

### Data analysis

The incoming responses were compiled and reviewed descriptively. Responses with similar content were grouped and organized under thematic headings. These themes were used to structure the presentation of the results. The consensus rate was calculated as a percentage.

### Ethical considerations

This study received ethical approval from the Swedish Ethical Review Authority on 15 May 2025 (Dnr: 2025-02620-01). All participants were informed about the purpose of the study, procedures for data handling and data protection, anonymity, confidentiality and their right to withdraw at any time. Informed written consent to participate and for publication was obtained from all participants.

## Results

All item-level changes across Delphi rounds are documented in Supplementary Table S1, providing a transparent audit trail from the initial 39 items to the final 29-item instrument.

### Round 1: Expert group

Experts assessed the initial 39 items for clarity and relevance. Their qualitative feedback identified four main areas requiring modification. Terminology: several medical terms (e.g., perimenopause, urogenital symptoms, osteoporosis) were challenged. Some noted that menopause-related terms may be unfamiliar to many respondents, which could compromise comprehension. Experts expressed differing opinions on how much technical language would be reasonable to expect. Language accessibility: experts recommended replacing complex terms with more accessible wording. Some terminology was replaced with more accessible alternatives. Clarity: most knowledge and symptom-related items were considered generally clear. Relevance: experts suggested adding two items concerning progesterone and testosterone. Based on the expert suggestions, 27 items were revised.

### Round 2: Public and Patient Involvement (PPI) group

The PPI-group contributed feedback in a workshop and through the online survey. Their feedback focused on five main areas. Readability challenges: participants expressed concern about terms such as perimenopause, postmenopause, evidence-based, progesterone, MHT and risks. Cognitive load: several participants reported fatigue or loss of focus due to survey length and cognitive demand. Relevance: all participants affirmed that the questions were perceived as relevant and appropriate. Terminology management: participants highlighted the difficulty of simplifying medical terminology while retaining accuracy. They recommended retaining correct terms but adding simpler alternatives in parentheses and ensuring the availability of a “Don’t know” response option, which was subsequently incorporated into the questionnaire. Risk-framing: participants noted that wording related to MHT and risks, particularly numerical risk comparisons, could be anxiety-provoking or reinforce existing misconceptions. These items were subsequently reworded to reduce the risk of amplifying fear. No items were added or removed in this round, but issues raised informed later revisions and item prioritization.

### Round 3: Expert group

Eight of the nine experts submitted their response in the prioritization round. Their feedback highlighted several issues related to prioritization of items to be included in the questionnaire. Bias in framing: some experts emphasized that items related to hormone therapy required more neutral phrasing. Risk-framing: consistent with the PPI feedback, experts noted that numerical risk formulations or comparisons involving breast cancer could unintentionally reinforce misconceptions or provoke fear. Terminology concerns: experts again discussed challenges related to technical terminology. Some items were deemed too medically dense to allow fair assessment for a general population. Survey length and overall burden: several items were recommended for removal due to redundancy or because they relied on terms that would require substantial prior knowledge. Applying the predetermined consensus rule resulted in 9 items being removed, 5 items merged into 2 items and 1 item added.

To address concerns about fear and misunderstanding highlighted in round 2 and 3, the research team developed a contextual introduction with a glossary and a detailed after-text providing evidence-based information and clarifying common myths. These additions were based on direct feedback from experts and formed part of the item refinement for subsequent rounds.

### Round 4: Pilot test group

The 48 pilot test group members completed the pilot test. Their feedback focused on several areas. Usability and completion time: 83% completed the survey in under 30 minutes. Engagement: 70% found the survey interesting. Self-perceived knowledge gap: 58% reported discovering gaps in their own menopausal knowledge during completion. Terminology: qualitative feedback on the survey showed that terms regarding MHT and hormones were difficult, especially for those who had not been in contact with them; using medical terms presupposes knowledge that some respondents may not have. Optional MHT section: based on user feedback, a group of MHT-related questions were changed to being optional. This allowed respondents who were uncomfortable or unfamiliar with the terminology to opt out, thereby reducing cognitive burden and preserving fairness in assessing knowledge. One item was removed.

### Round 5: Expert group

Eight experts participated in the final approval round. Consensus was achieved with an 87.5% agreement. One expert suggested a minor revision, which was incorporated into the final version of the questionnaire. Following content and face validation, the final instrument comprised 29 items.

Key qualitative feedback from experts, PPI members, and pilot test participants was analyzed thematically. A summary of the main themes and illustrative quotes across Delphi rounds is presented in Table 3.

**Table 3.** Summary of key qualitative feedback across Delphi rounds.

Theme	Source (round)	Illustrative quote (translated from Swedish)
Medical language & terminology	Expert group Round 1	“Menopause is clearly defined, as are pre-, peri-, and postmenopause; it would be clearer to use those terms.”
	Expert group Round 3	“Menopause itself is a phase, so I think we do not need to talk about perimenopause when we have the Swedish term, it becomes confusing.”
	PPI Group Round 2	“Keep the correct medical words but put explanations in brackets. We want to learn.”
Clarity & readability	Expert group Round 1	“What is meant by long-term health effects? How is it connected to menopause? Adapt to the target group.”
	Expert group Round 3	“It is enough to write after menopause, again, do not complicate it with more concepts for either women or healthcare staff.”
	Pilot test group Round 4	“Medical terms should be avoided.”
Risk framing & fear	PPI Group Round 2	“Being over 60, I now worry, since I didn´t seek healthcare for my menopausal symptoms… how will this impact my long-term health? “
	Expert group Round 1	“Another good example that illustrates risk in an everyday context.”
	Expert group Round 3	“Numerical comparisons about breast cancer risk may unintentionally reinforce fear.”
Bias & neutrality	Pilot Test group Round 4	“I experience the questionnaire as being against systemic hormone therapy.”
	Expert group Round 1	“I would prefer to write ‘there is probably no increased risk.’”
	Expert group Round 1	“‘No action is needed; it is normal and will pass’ – an unnecessary value judgment?”
Cognitive burden & length	Expert group Round 1	“For someone who is familiar with informed choice, all questions are interesting, but if you are not, it may feel like too much.”
	PPI Group Round 2	“Now I am starting to feel that there are maaaany questions.”
	Expert group round 5	“There is a risk, according to me that the participants don´t understand the questions and therefore don´t answer.”
Health literacy needs	PPI Group, Round 2	“Evidence-based, stigma, and systemic may need to be explained.”
	Expert group Round 3	“It needs to be ensured that the respondent understands what systemic hormone therapy is, otherwise, the question is not valid or reliable.”

*Note.* All qualitative comments were originally provided in Swedish. Illustrative quotes were translated into English and kept as close as possible to the original wording.

## Discussion

This study used a modified Delphi approach to develop and establish content and face validity of a menopause-specific questionnaire designed to assess women’s access to the preconditions for making informed choices about their health during their menopausal transition. By integrating expertise from participants with lived experiences, the resulting instrument establishes face validity as well as contextual relevance for its target population.^30,33^

### Overall Delphi panel engagement and participatory co-construction

The involvement of expert group members, PPI group members, and pilot group members strengthened the depth and relevance of the questionnaire. The iterative design ensured that linguistic precision, conceptual clarity, and perceived relevance were continuously improved. The inclusion of lived experience, both in the PPI and pilot test groups, was particularly valuable for evaluating language accessibility, respondent burden and clarity of the items. As concluded by Rolstad et al.,^20^ respondent burden is not primarily tied to time to complete survey but rather by experienced relevance and clarity of the content. The overall Delphi Panel members feedback guided several design modifications, including the addition of a glossary and contextual introduction, an optional MHT knowledge section to reduce cognitive burden, and an after-text containing evidence-based information to mitigate misconceptions and support informed choice. These adjustments improved clarity and accessibility and reduced cognitive burden while maintaining the integrity of the consensus-building process. Although the panel lacked representation from certain stakeholder groups, such as general practitioners and more diverse community perspectives, the heterogeneity of the experts created multi-layered engagement that contributed substantially to the content validity of the final instrument.^29^ The use of digital, asynchronous expert group rounds enabled convenient participation and reduced the risk of dominance by more vocal individuals or hierarchical influences that may arise in in-person processes.^36^ At the same time, this format limited opportunities for spontaneous dialogue which could have enriched item interpretation.^29^ To mitigate this, later rounds incorporated a short video briefing alongside written instructions, which improved members’ comprehension and engagement. In a field with limited validated instruments, this participatory approach allowed for the synthesis of diverse types of knowledge, supporting the development of a questionnaire that is operationally feasible within the Swedish context and conceptually adaptable for use in other settings pending cultural and linguistic validation.

### Methodological challenges: Terminology, knowledge assessment, and avoiding reinforcing fear

Developing a menopause-specific knowledge instrument presented several methodological challenges. A central issue concerned the use of menopausal terminology. Across both expert and PPI group rounds, members disagreed on whether medically precise terms, such as perimenopause, postmenopause, and terminology related to MHT, should be used. While some emphasized the importance of accuracy, others argued that these terms are unfamiliar to many women and risk reducing comprehension and response accuracy. This tension emerged not between groups but within them, reflecting broader inconsistencies in how menopausal concepts are understood across clinical practice, public discourse, and everyday language. The difficulty in agreeing on the nomenclature in women’s sexual and reproductive health, and how that impacts health literacy, is highlighted by Critchley et al.^19^ describing challenges in the menstrual context, where limited accessible information and inconsistent terminology may restrict women’s ability to communicate with healthcare professionals and to make informed decisions about their health. These methodological challenges led to the removal or revision of several items that relied on terminology considered too complex for fair assessment. To further address these challenges, the researchers incorporated multiple health-literacy–supportive strategies, including a contextual introduction with a glossary, simplified wording in brackets, and an optional section on MHT knowledge for respondents who felt comfortable engaging with more technical material. This approach was guided by principles of health literacy and informed choice, particularly given the limited Swedish lay language regarding menopause.^37^ The MHT knowledge items were developed in accordance with the Swedish national guidelines on menopausal healthcare,^38^ which represent the current evidence-based standard for clinical practice in Sweden. Khandehroo et al.^39^ showed that educational interventions to improve menopausal health literacy lead to improved life quality among menopausal women in their study. By retaining medically accurate terms such as perimenopause, and pairing them with clear explanations, the questionnaire supports both immediate understanding and the development of the vocabulary women need to communicate effectively about their health.

A second methodological challenge involved addressing breast cancer risk and MHT without prompting fear or reinforcing common misconceptions. Some questionnaire items explore women’s perceptions of menopausal symptoms and effects of MHT, and therefore include options not fully supported by current evidence. These were intentionally included to capture knowledge gaps and misconceptions. To avoid reinforcing misinformation, participants received evidence-based after-text following completion of the questionnaire, ensuring that the instrument captures existing beliefs rather than reinforcing them. Both expert and PPI group members expressed concern that numerical risk formulations and comparative statements could unintentionally amplify anxiety. And although topics such as breast-cancer risk and MHT may provoke worry, evidence from decision-aid research shows that providing clear risk information is essential for informed choice and should not be excluded solely because it may trigger emotional responses.^40^ Accordingly, the research team undertook substantial revisions, including the removal of some items and careful rephrasing of others. To balance sensitivity with accuracy, the contextual introduction and a detailed after-text providing evidence-based information and clarifications of common myths were added, based on the guidelines from The Swedish Society of Obstetricians and Gynecologists.^38^ These additions allowed sensitive topics to remain in the questionnaire while reducing the likelihood of misinterpretation or heightened worry. The after-text also served a secondary purpose by increasing participants’ menopausal knowledge, aligning with the broader aims of the research project. Together, these methodological challenges illustrate the complexity of developing a menopause-specific instrument in a context marked by inconsistent terminology, widespread misconceptions, and strong emotional associations, particularly regarding breast cancer and MHT. These challenges, as evidenced in expert and PPI group members’ feedback, highlight the importance of iterative, participatory processes that balance scientific accuracy, readability, and ethical communication.

### Feasibility and usability of the questionnaire

Revisions across the Delphi rounds addressed clarity, accessibility, and cognitive burden as reflected in participant feedback. Simplified wording and reduced reliance on specialist terminology made the questionnaire more usable for a broad demographic. The move from externally developed instruments to a participatory, co-constructed approach strengthened both contextual relevance and local ownership. By centering women’s lived experiences throughout development, following content and face validation, the questionnaire reflects both public and personal dimensions of the menopausal experience, supporting its feasibility for real-world application.

### Validity and transferability

The content validity of the Delphi results is supported by the iterative, structured consensus-building process and by the experts. The PPI and pilot test groups provide face validity through target audience and lived experience perspectives. Transferability is strengthened by the multidisciplinary composition of the expert panel and by the inclusion of women with diverse menopausal experiences.^29^ At the same time, transferability is limited by the relatively small expert panel size and the Swedish-language context, which may restrict generalization to settings with different healthcare systems or linguistic environments. Nonetheless, the core domains identified, menopausal knowledge, attitudes and beliefs, and access to healthcare, are conceptually transferable and relevant to wider clinical and public health contexts.

### Strengths and limitations

A key strength of this study is its integration of established theoretical frameworks. Drawing on validated tools, MMIC, HLQ, and DCS/SURE provided strong psychometric grounding and allowed for the structuring of items into coherent domains such as knowledge, attitudes, and healthcare access. The modified Delphi design, which incorporated lived experience at several stages, represents an innovative approach seldom used in questionnaire development yet crucial for ensuring person-centered relevance.^30^ Including both academic experts, PPI-group and pilot test participants provided different perspectives and nuances, all equally important to the tool development. Furthermore, the interdisciplinary research group enabled critical discussions involving different perspectives during the process.

Several limitations must be acknowledged. The expert group was small (n=9 round 1, n=8 rounds 3-5), and the design, which protected respondent confidentiality, meant that the research team could not directly identify or follow up with non-respondents. The impact of a single dropout on consensus percentages is therefore non-trivial. Nevertheless, the panel’s composition across academic, clinical, advocacy, and lived-experience perspectives is consistent with Delphi recommendations for purposively selected, information-rich panels, where heterogeneity of expertise and experience is prioritized over sample size.^29,36^ Only eight experts responded in Rounds 3 and 5, raising the possibility that the non-respondent expert did not agree with the direction of the questionnaire. Because several items address medically sensitive content, such as MHT and breast-cancer risk, adequate clinical representation on the panel was important. As responses were collected confidentially, the research team could not determine whether the non-responding expert in Rounds 3 and 5 was one of the three gynecologists (MDs) on the panel; the clinical accuracy of these items was nonetheless supported by the continued participation of multiple gynecologists and by anchoring them to the Swedish national guidelines on menopausal care. Asynchronous communication limited opportunities for dynamic dialogue. While the PPI group participated in a face-to-face workshop, which is a common approach in patient and public involvement,^33^ this format introduces potential social conformity bias. To reduce this risk, the workshop was moderated to encourage equal participation, and PPI members were additionally invited to provide individual written feedback independently of the group setting. Nevertheless, the potential influence of group interaction is acknowledged as a methodological limitation. The underrepresentation of general practitioners and minority groups restricts the breadth of perspectives captured, and the Swedish-only format of the pilot test limits linguistic and cultural generalizability. Although the pilot population was relatively highly educated, which may limit immediate insight into how the questionnaire performs among individuals with lower health literacy, the instrument was specifically developed to address known barriers to informed choice across diverse populations. Research shows that women with lower socioeconomic status or limited health literacy often face challenges in accessing reliable information, navigating healthcare systems, and overcoming stigma and misinformation related to menopause.^1,18^ Several design features were therefore incorporated to enhance accessibility, including simplified language, glossary support, and contextual explanations - strategies recommended for improving comprehension across varying literacy levels^35^. The inclusion of both knowledge and perception-based items allows the questionnaire to identify disparities in understanding as a prerequisite for informed decision-making.^13,28^ Future validation studies should include more socioeconomically diverse populations to further examine the instrument’s applicability where barriers to informed choice are most pronounced.

These limitations should be considered when interpreting the scope and applicability of the instrument. Psychometric reliability testing is not within the scope of this study^36^ and is recommended for future validation studies.

Some Delphi Panel members had professional relationships with members of the research team. Although these relationships were openly acknowledged, a reflexive approach and clear communication about roles and boundaries were implemented to reduce potential influence on the process or outcomes.

### Future directions

The questionnaire will be distributed in a cross-sectional survey study in Sweden starting 2026, available in Swedish and English. The results from the questionnaire have the potential to inform clinical practice and guide health education. It may inform policy development aimed at improving access to evidence-based care and enhancing women’s ability to make informed choices about their health throughout their menopausal transition and beyond. Future iterations would benefit from broader stakeholder involvement and testing to strengthen the questionnaire’s validity and applicability. Psychometric validation, including assessment of internal consistency, construct validity, and test-retest reliability, is recommended as a necessary next step before large-scale implementation.

## Conclusion

This study established content and face validity of a menopause-specific questionnaire that provides a structured and participatory tool for assessing women’s access to the preconditions necessary for making informed choices about their health during the menopausal transition. By identifying knowledge gaps, misconceptions, and barriers to healthcare, this questionnaire lays a foundation for woman-centered interventions that enhance informed choice, understanding of menopause, and awareness of treatment options. The questionnaire can support the identification of areas where targeted interventions may improve understanding, access to care, and decision-making.

## Supplemental material

Supplemental material - Development of a menopause-specific questionnaire: Content and face validation using a modified Delphi technique Supplemental material for Development of a menopause-specific questionnaire: Content and face validation using a modified Delphi technique by Amanda Calvin, Camilla Udo, and Kerstin Erlandsson in Women’s Health.

## Data Availability

All relevant data available in supplementary files.*
