# Supplementary material for: Development of a menopause-specific questionnaire: Content and face validation using a modified Delphi technique
Source: Womens Health (Lond). 2026 Aug 4;22:17455057261476566. doi: 10.1177/17455057261476566 (PMC13438354; doi:10.1177/17455057261476566)
Supplement: Supplementary file 1 [file sj-pdf-1-whe-10.1177_17455057261476566.pdf]

Supplementary Table S1. Audit trail of item development across Delphi rounds (R1–R5)

The table documents item-level revisions, deletions, and final status throughout the modified Delphi process.

Original item (Round 1 wording)	Origin	Domain	Delphi round(s) of change	Summary of feedback and revisions (R1–R5)	Final item wording	Final status
Q1. What is menopause?	MMIC	Knowledge / Education	R1	R1: Rephrasing, added answering options	Which of the following statements about menopause do you believe are correct?	Revised and retained
Q2. Which of the following statements do you believe are true about perimenopause?	MMIC	Knowledge / Education	R1	R1: Rephrasing, added answering options	Which of the following statements do you believe are correct about perimenopause?	Revised and retained
Q3. Which of the following statements do you believe are true about postmenopause?	MMIC	Knowledge / Education	R1	R1: Rephrasing, added answering options	Which of the following statements do you believe are correct about postmenopause?	Revised and retained
Q4. How many women do you think experience menopausal symptoms?	MMIC	Knowledge / Education	R3	R3: Deleted		Removed
Q5. How many do you think experience moderate to severe symptoms?	MMIC	Knowledge / Education	R1, R3	R1: Rephrasing | R3: Deleted		Removed
Q6. Which of the following do you think can be menopausal symptoms?	MMIC	Knowledge / Education	R3	R3: Clarification	Which of the following do you think can be symptoms of menopause?	Revised and retained
Q7. In your view, which long-term health effects may be connected to menopause?	MMIC	Knowledge / Education	R1, R3	R1: Rephrasing, added answering options | R3: Deleted		Removed
Q8. Which of the following statements about menopause do you believe are correct?	MMIC	Knowledge / Education	R1, R3, R4	R1: Added answering option | R3: Rephrasing and clarification | R4: Removal of answering options and rephrasing	Which of the following statements about menopause do you believe are correct?	Revised and retained
Q9. Which of the following do you think are evidence-based long-term treatments for menopausal symptoms?	MMIC	Knowledge / Education	R1, R3	R1: Clarification of answering option and added option | R3: Clarification	Which approaches to managing menopause symptoms do you believe have scientific support?	Revised and retained
Q10. How effective is hormone therapy compared with other treatments for hot flashes and night sweats?	MMIC	Knowledge / Education	R1, R3	R1: Clarification | R3: Deleted		Removed

Q11. How safe is hormone therapy for healthy women aged 40–50 with menopausal symptoms?	MMIC	Knowledge / Education	R1, R3, R4	R1: Clarification | R3: Rephrasing and clarification | R4: Deleted		Removed
Q12. Which of the following statements do you believe are true about local (vaginal) estrogen therapy?	MMIC	Knowledge / Education	R1, R3	R1: Clarification of answering option and added option | R3: Rephrasing, clarification, removal of answering options	Which of the following do you believe are true about local vaginal estrogen?	Revised and retained
Q13. Which of the following reasons best match why you might consider hormone therapy?	MMIC	Attitudes	R3, R4	R3: Rephrasing | R4: Rephrasing	Which of the following best describe your reasons for considering MHT?	Revised and retained
Q14. What was the single most important reason you chose to start hormone therapy?	MMIC	Attitudes	R1, R2, R3	R1: Rephrasing | R2: Added answering option | R3: Clarification	What was the single most important reason you chose to start MHT?	Revised and retained
Q15. Which reasons best match why you chose not to use hormone therapy?	MMIC	Attitudes	R3	R3: Clarification, added answering option	What is your main reason for choosing not to use MHT?	Revised and retained
Q16. According to your understanding, which symptoms or conditions can hormone therapy reduce, relieve, or prevent?	MMIC	Knowledge / Education	R5	R5: Which symptoms or conditions do you believe MHT can help reduce, relieve, or prevent?	Which symptoms or conditions do you believe MHT can help reduce, relieve, or prevent?	Revised and retained
Q17. If 100 women aged 50–60 do NOT use hormone therapy, about 3 will get breast cancer. If these 100 women instead use hormone therapy for at least 5 years, how many MORE do you think would develop breast cancer?	MMIC	Knowledge / Education	R1, R2, R3	R1: Clarification | R2: Clarification and added answering option | R3: Deleted		Removed
Q18. What do you think an increased breast cancer risk from hormone therapy means?	MMIC	Knowledge / Education	R3, R4	R3: Rephrasing, removal of answering options | R4: Change question format. Removal of answering options	I believe the risk of breast cancer with long-term combined MHT is:	Revised and retained
Q19. If 100 women aged 50–60 use combined hormone therapy (estrogen + progesterone/progestogen) for 5 years, how do you think breast cancer mortality changes over the next 10 years?	MMIC	Knowledge / Education	R1, R3	R1: Clarification | R3: Deleted		Removed

Q20. Which of the following do you think carry the same or higher breast cancer risk as using combined hormone therapy for 5 years?	MMIC	Knowledge / Education	R1, R3	R1: Rephrasing and clarification | R3: Removal of answering option	Which of the following do you believe increase breast cancer risk as much as or more than long-term combined MHT?	Revised and retained
Q21. If you had (or imagine having) bothersome menopausal symptoms, which primary approach would you choose?	MMIC	Attitudes	R1, R3	R1: Added answering option | R3: Clarification	If you have menopausal symptoms now, or imagine having them in the future, what primary treatment approaches would you consider?	Revised and retained
Q22. Think about the option you prefer and answer the following about your menopausal health and care:	SURE + DCS	Healthcare Access & Support	R1, R3	R1: Rephrasing | R3: Merged 22+27+28. Deleted answering options. Rephrasing	Please state to what degree you agree with the following statements:	Removed
Q23. I would likely find a personal consultation with a healthcare provider about menopause to be:	MMIC	Attitudes	R3	R3: Rephrasing	I would likely find a personal consultation with a healthcare professional about menopause to be:	Revised and retained
Q24. My menopausal health is:	MMIC	Attitudes	R1	R1: Rephrasing	How much of a priority is your long-term health in relation to menopause?	Revised and retained
Q25. My attitude toward menopause is:	MMIC	Attitudes	R3	R3: Deleted		Removed
Q26. My trust in menopause-related healthcare is:	MMIC	Attitudes	R3	R3: Deleted		Removed
Q27. Check the option that best matches your experience:	HLQ	Healthcare Access & Support	R1, R3	R1: Rephrasing | R3: Merged 22+27+28. Deleted answering options. Rephrasing		Removed
Q28. Choose the option that best matches your experience:	HLQ	Healthcare Access & Support	R1, R3	R1: Rephrasing | R3: Merged 22+27+28. Deleted answering options. Rephrasing		Removed
Q29. To what extent do you agree with the following statements about hormone therapy?	MMIC + HLQ	Attitudes	R1, R3, R4	R1: Rephrasing | R3: Rephrasing and clarification | R4: Rephrasing	To what extent do you agree with the following statements about MHT?	Revised and retained
Q30. Have you discussed treatment options for menopause (hormone therapy, natural remedies, lifestyle changes) with a healthcare provider?	HLQ	Healthcare Access & Support	R3	R3: Clarification	Have you discussed treatment options for menopausal symptoms with a healthcare professional?	Revised and retained
Q31. Have you experienced any barriers to receiving menopause-related care or information?	HLQ-inspired + developed	Healthcare Access & Support	R1, R2, R3	R1: Added answering option | R2: Rephrasing | R3: Rephrasing, clarification, added answering options	Have you experienced any barriers when seeking care for menopausal symptoms?	Revised and retained

Q32. Preferences for discussing menopause:	HLQ + developed	Healthcare Access & Support	R1, R3	R1: Added answering option | R3: Merged 32+39. Change question format	I feel comfortable discussing menopause with:	Revised and retained
Q33. Read the statements below and tick the box that best describes how you feel right now:	MMIC	Attitudes	R1, R2, R3, R4	R1: Clarification | R2: Rephrasing | R3: Rephrasing | R4: Rephrasing	Please read the statements below and select the option that best describes how you currently feel about menopause. There are no right or wrong answers. Try to respond spontaneously.	Revised and retained
Q34. I think menopause is/has:	Developed	Attitudes	R3	R3: Removal of answering options | R5: I believe menopause is:	I believe menopause is:	Revised and retained
Q35. What is your primary source of information about menopause?	HLQ-inspired	Knowledge / Education	R1	R1: Added answering option | R5: What is your primary source of information about menopause?	What is your primary source of information about menopause?	Revised and retained
Q36. To what extent do you agree with the following statements about menopause?	HLQ + developed	Knowledge / Education	R1, R3	R1: Clarification | R3: Removal of answering options	To what extent do you agree with the following statements:	Revised and retained
Q37. Do you think menopause education should be improved in Sweden?	Developed	Attitudes	R1, R3	R1: Rephrasing | R3: Deleted		Removed
Q38. When should menopause education be provided?	Developed	Knowledge / Education	R2, R3, R4	R2: Rephrasing | R3: Added answering option. Clarification | R4: Clarification	When do you think information about menopause should be offered?	Revised and retained
Q39. From whom would you prefer to receive information about menopause?	Developed	Knowledge / Education	R1, R3	R1: Added answering option | R3: Merged 32+39	Q39. From whom would you prefer to receive information about menopause?	Revised and retained
Q40. Which of the following do you believe are true about testosterone?	Expert suggestion (R1)	Knowledge	R1, R3	R1: New question: R3: Added answering option	Which of the following do you believe are true about testosterone?	Revised and retained
Q41. Which of the following do you believe are true about progesterone?	Expert suggestion (R1)	Knowledge	R1, R3	R1: New question: | R3: Removal of answering option. Clarification	Which of the following do you believe are true about progestogens?	Revised and retained
Q42. Did you have any questions about menopause that were not answered during the health contact?	Expert suggestion (R3)	Healthcare Access & Support	R3	R3: New question	Did you have any questions about menopause that were not answered during that healthcare contact?	Revised and retained
